# Influence of organic molecules on the aggregation of TiO_2_ nanoparticles in acidic conditions

**DOI:** 10.1007/s11051-017-3807-9

**Published:** 2017-04-04

**Authors:** Karin Danielsson, Julián A. Gallego-Urrea, Martin Hassellov, Stefan Gustafsson, Caroline M. Jonsson

**Affiliations:** 1 Department of Chemistry and Molecular Biology, University of Gothenburg, 412-96 Gothenburg, Sweden; 2 Department of Marine Sciences, University of Gothenburg, 412-96 Gothenburg, Sweden; 3 Department of Applied Physics, Chalmers University of Technology, 412-96 Gothenburg, Sweden

**Keywords:** TiO_2_ nanoparticles, Anatase, Aggregation, Adsorption, Organic molecules, ζ-potential, Environmental effects

## Abstract

Engineered nanoparticles released into the environment may interact with natural organic matter (NOM). Surface complexation affects the surface potential, which in turn may lead to aggregation of the particles. Aggregation of synthetic TiO_2_ (anatase) nanoparticles in aqueous suspension was investigated at pH 2.8 as a function of time in the presence of various organic molecules and Suwannee River fulvic acid (SRFA), using dynamic light scattering (DLS) and high-resolution transmission electron microscopy (TEM). Results showed that the average hydrodynamic diameter and ζ-potential were dependent on both concentration and molecular structure of the organic molecule. Results were also compared with those of quantitative batch adsorption experiments. Further, a time study of the aggregation of TiO_2_ nanoparticles in the presence of 2,3-dihydroxybenzoic acid (2,3-DHBA) and SRFA, respectively, was performed in order to observe changes in ζ-potential and particle size over a time period of 9 months. In the 2,3-DHBA-TiO_2_ system, ζ-potentials decreased with time resulting in charge neutralization and/or inversion depending on ligand concentration. Aggregate sizes increased initially to the micrometer size range, followed by disaggregation after several months. No or very little interaction between SRFA and TiO_2_ occurred at the lowest concentrations tested. However, at the higher concentrations of SRFA, there was an increase in both aggregate size and the amount of SRFA adsorbed to the TiO_2_ surface. This was in correlation with the ζ-potential that decreased with increased SRFA concentration, leading to destabilization of the system. These results stress the importance of performing studies over both short and long time periods to better understand and predict the long-term effects of nanoparticles in the environment.

**Electronic supplementary material:**

The online version of this article (doi:10.1007/s11051-017-3807-9) contains supplementary material, which is available to authorized users.

## Introduction

Nanotechnology is a rapidly growing industry and an increased amount of synthetic nanoparticles is released into the environment (Keller et al. 2010; Roco 2011; Vance et al. 2015). Nanoparticles are defined as objects having at least one dimension smaller than 100 nm, and they generally have higher reactivity than larger particles of the same material due to a much higher specific surface area and abundance of active sites (Bottero et al. 2011; Hotze et al. 2010; Wiesner et al. 2006). These properties may enhance processes like dissolution, redox catalytic activity, and ligand sorption capacity and strength, which ultimately affect the toxic response (Hochella et al. 2008). Nanoparticles may interact with natural organic matter (NOM), such as humic and fulvic acids, which is present in most natural waters. Thus, titanium dioxide (TiO_2_) nanoparticles released into surface waters might be coated with these molecules, leading to the formation of a corona that may alter the properties of the nanoparticles. Adsorption of NOM affects the surface speciation and net charge of the particles and is therefore of great importance for their colloidal stability. This might alter the mobility of nanoparticles in surface waters and in soils, thus determining their behavior, fate, transport, and bioavailability. It is therefore of great concern to elucidate the molecular interaction processes between nanoparticles and organic molecules in aquatic environments (Bian et al. 2011; Thio et al. 2011; Yang and Cui 2013). For example, Tiller et al. reported enhanced stability of hematite particles in suspensions containing NOM (Tiller and O’Melia 1993).

In aqueous suspension, metal oxide nanoparticles have surface sites that are protonated or deprotonated depending on pH. This gives rise to a surface potential that is further balanced by counter ions in aqueous solution, and this is referred to as the electrical double layer (EDL) (Stumm 1993). An increase in ionic strength will compress the EDL and the van der Waals attractive forces will become higher than the electrostatic repulsion forces and aggregation will occur (Atmuri et al. 2013; Fokkink et al. 1987). According to the DLVO theory (Atmuri et al. 2013; Petosa et al. 2010), the surface potential, which is a forcing factor for nanoparticle stability and aggregation behavior in aqueous suspension, is strongly dependent on pH and ionic strength. Hence, the mineral phase-specific surface potential is highly affected by the surrounding media.

When pH is close to the pH_PZC_ (point of zero charge), the net surface potential is close to 0 and the electrostatic repulsion is low. Consequently, particles aggregate to form larger colloids (Grassian 2008). ζ-potential is the electric potential that develops at the surface of the shear, i.e., the potential difference between the stationary layer of fluid at the surface and the potential in the bulk medium. Hence, the ζ-potential depends on the surface charge, as well as the composition of the surrounding media, such as pH, type of counter ions, and ionic strength. ζ-potential is a widely used index to measure the colloidal stability of particle dispersions (Liao et al. 2009).

Organic molecules adsorb to particle surfaces through electrostatic and/or specific interactions with particle surface sites, although hydrophobic interactions might also contribute to the adsorption of large macromolecules, such as NOM (Tiller and O’Melia 1993). Charges accumulate as a result of surface complexation between surface sites and species in solution. Organic ligands can be associated with the surface either in an inner- or outer-sphere fashion, or a combination of the two depending on the type and position of available surface sites, the molecular structure of the ligand and presence of functional groups, and suspension pH (Huang et al. 1995). Specific adsorption results in inner-sphere complexes containing a direct bond between the ligand and the surface metal. Outer-sphere complexes, on the other hand, are mainly stabilized by electrostatic attractions and/or hydrogen bonding and are formed when one or several water molecules are present between the ligand and the surface metal, which leads to an accumulation of ligands in the EDL. Organic molecules that contain several functional groups may have several points of attachment to the surface, thus involving both inner-sphere and outer-sphere bonding at the same time. This was previously shown to be the case for the amino acids glutamic and aspartic acid, respectively, binding with one or both carboxyl groups to microsized titanium dioxide (rutile) particles in aqueous solution. The mode of attachment was shown to be strongly dependent on both pH and ligand-to-solid ratio (Jonsson et al. 2010; Jonsson et al. 2009; Parikh et al. 2011).

A similar reasoning is expected to be appropriate for the humic fraction present in NOM, consisting of high molecular weight organic molecules, such as humic and fulvic acids. NOM molecules are likely branched, thus not linear or flexible, and may have substantial hydrophobic character. Nevertheless, they contain a high abundance of functional groups, such as phenolic, carboxylic, and hydroxyl groups all prone to bind to metal oxide surfaces in water. Kleber et al. suggests a model where soil organic matter (SOM) sorbs to mineral surfaces in zones located at different distances from the mineral surface (Kleber et al. 2007). In the contact zone, an organic molecule with charged functional groups adsorbs specifically to mineral surface sites, leaving the hydrophobic part of the molecule pointing away from the surface. This hydrophobic part might interact with the hydrophobic part of a second organic molecule, resulting in a membrane-like bilayer of sorbed ligands in the hydrophobic zone. The polar part of the second molecule points towards the aqueous phase in the outer kinetic zone, hence, the hydrophobic moieties of sorbed molecules are protected from contact with the aqueous solution. The polar parts of molecules in the kinetic zone are free to interact with ions or other organic molecules in suspension, which might create additional layering (Kleber et al. 2007). pH and ligand-to-solid ratio are likely to determine the extent of surface coverage and mode of attachment, which will in turn affect the surface potential. Further, pH will affect the thickness of the adsorbed layer due to (de) protonation of loops and tails on the NOM molecules as they are bound to the particle surface. Interactions between particles are dependent on interactions between the layers of adsorbed organic molecules (Tiller and O’Melia 1993) and this will influence the colloidal stability, since surface complexation may lead to aggregation by breaking the balance between the electrostatic, hydrophobic, and steric forces. While complexed at the surface, organic molecules may adjust their conformation in response to changing conditions in suspension, which emphasizes the importance of studying these processes as a function of time. Moreover, due to the complex nature of NOM molecules, it is useful to include studies of smaller organic molecules in order to model certain aspects of NOM, such as the influence of type and position of charged functional groups on the aggregation behavior of TiO_2_ nanoparticles. Surface complexation of ligands often contributes to a change in the surface potential through the formation of charged surface complexes, which is crucial for the colloidal stability.

TiO_2_ nanoparticles are widely used in various applications and commercial products, such as additives in paints, sunscreens, cosmetics, solar cells, plastics, etc. (Menard et al. 2011), and this material has been subjected to previous investigations of fate and toxicity (Domingos et al. 2009; Loosli et al. 2013; von der Kammer et al. 2010). Thio et al. studied the colloidal stability of TiO_2_ in NaCl or CaCl_2_ electrolytes and found that Suwannee River humic acid (SRHA) drastically increased the stability of TiO_2_ nanoparticles, which might be due to increased electrostatic and steric repulsions (Thio et al. 2011). Loosli et al. showed how the interactions of SRHA and TiO_2_ nanoparticles were affected by changes in pH at a fixed concentration of 100 mg/L SRHA. High pH (pH = 11) led to very high negative ζ-potentials (∼ − 70 mV), and charge inversion was obtained by varying the concentration of SRHA at pH 4.5 (Loosli et al. 2013). These results were in agreement with Thio et al. (2011), although their study was performed at pH ∼5 which is closer to environmental relevant conditions but also very close to the pH_PZC_. Consequently, particles might not have been stable at the beginning of the experiment, which makes it somewhat difficult to interpret the results. von der Kammer et al. (2010) showed in a similar study that charge reversal was obtained after addition of CaCl_2_ to the TiO_2_ nanoparticle suspension. Pettibone et al. (2008) investigated the aggregation of TiO_2_ nanoparticles in the presence of various organic molecules and their results showed that aggregation with oxalic acid occurs at pH 2 and 6.5, and larger aggregates are formed with smaller particles (5 nm) than those with larger ones (32 nm). Molina et al. (2011) studied amino acid interactions with ZnO nanoparticles and found that the amino acid concentration and solution pH, as well as the type of amino acid tested, affect the aggregation of ZnO nanoparticles. Gallego-Urrea et al. (2014) studied the kinetics of electrolyte-induced aggregation of TiO_2_ NP coated with different NOMs and found that pH and the type of organic macromolecule induced contrasting differences in the aggregation behavior.

The objective of the present work was to improve the understanding of the colloidal stability of synthetic nanoparticles by investigating the aggregation behavior of synthetic TiO_2_ nanoparticles in aqueous suspension as a function of time in the presence of organic molecules. Synthesized and well-characterized TiO_2_ (anatase) nanoparticles were used as model nanoparticles (Abbas et al. 2011). Selected phenolic carboxylic compounds were used as model substances to mimic the interactions of nanoparticles with NOM, and standardized Suwannee River fulvic acid (SRFA, International Humic Substances Society (IHSS)) was used in order to compare with a more environmentally realistic model material. The phenolic-to-carboxyl ratio of SRFA is approx. 1:4 (Ritchie and Perdue 2003), which justifies the comparison of SRFA with smaller model molecules containing carboxyl and phenolic groups (Table 1) to investigate the importance of the type and position of these functional groups. The aggregation behavior, as well as the electrophoretic mobility of the particles, was studied by simultaneously monitoring the changes in particle size and ζ-potential during the reactions. This was compared to batch experiments in order to quantify the amount of organic ligand adsorbed to the TiO_2_ nanoparticles. Further, a time study was performed in order to observe changes in surface potential and particle size over a time period of several months, which adds new information useful for understanding the long-term effects of nanoparticles in aqueous environments. A similar study to the present one was performed by Lee et al. (2016) at which they examine the aggregation behavior of TiO_2_ (rutile) nanoparticles (195 nm) in the presence of SRHA in DI water over a time period of 12 h. They observed only small changes in ζ-potential and hydrodynamic diameter over time. It is worth noting that Lee et al. (2016) used a solid concentration of 5 mg/L, which is 20 times lower than that in the present study (104 mg/L). Additionally, their experiments were performed at pH 7, compared to 2.8 in the present one. Further, we kept the samples on a rotator to avoid sedimentation during equilibration, which was not the case in the Lee et al. (2016) study. For these reasons, and the fact that both aggregation of particles and the adsorption of organic ligands are generally dependent on both ligand-to-solid ratios and pH, the results of these two studies cannot be fully compared.

**Table 1:**
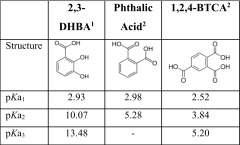
Molecular structures of organic model molecules with corresponding p*K*a values

^a^p*K*a_1_ and p*K*a_2_: determined at 0.01 M NaCl and 20 °C by potentiometric titrations in this laboratory (Rosenqvist and Jonsson 2017), p*K*a_3_: from various sources; proper reference missing

^b^Determined at an ionic strength of 0.03 M (Brown et al. 1955)

Besides the contribution to fundamental research, results from the present work may also be applicable on acid mine drainage sites worldwide, as well as other particular sites where pH is low.

## Experimental

### Chemicals and materials

TiO_2_ (anatase) nanoparticles used in this study were synthesized through a low-temperature-controlled hydrolysis of TiCl_4_ (Eq. 1).

1 $$ {\mathrm{TiCl}}_4 + {2\mathrm{H}}_2\mathrm{O}\Rightarrow {\mathrm{TiO}}_2+4{\mathrm{H}}^{+}+4{\mathrm{Cl}}^{\hbox{-} } $$

Due to the large amount of protons released during the synthesis, suspensions of formed TiO_2_ consist of nanoparticles of well-defined particle sizes stable at low pH (pH 2.8), thus are positively charged. The particles were characterized using dynamic light scattering (DLS), electrospray-scanning mobility particle sizing (ES-SMPS), X-ray diffraction (XRD) and transmission electron microscopy (TEM). Particles are “sphere-like” and consist predominantly of anatase (>92%). The size of the primary particles was found to be 4–5 nm irrespective of the particle size in suspension. Larger particles are formed due to aggregation of primary particles (Abbas et al. 2011). Particles used in the present study had an initial diameter of 13.3 ± 1.3 nm and a specific surface area of 206 m^2^/g that was determined using the BET N_2_ adsorption method (Brunauer et al. 1938). The pH_IEP_ was determined to be around pH 6 using ζ-potential titrations (Gallego-Urrea et al. 2014), and the pH_PZC_ was calculated to be between 6 and 6.2 depending on particle size using the CDH-SC theory (Holmberg et al. 2013).

2,3-Dihydroxybenzoic acid (2,3-DHBA; 99%), phthalic acid (99.5%), and 1,2,4-benzenetricarboxylic acid (1,2,4-BTCA; 99%) were purchased from Sigma Aldrich and used without further purification (Table 1). SRFA (2S101F) was obtained from the IHSS. The experimentally calculated molecular weight of SRFA is 551 ± 10 Da (Fattahi and Solouki 2003); hence, the molecular weight used for calculating molar concentrations in the present work was 551 g/mol. SRFA molecules have a hydrodynamic diameter between 1.5 and 2.5 nm (Hassellov et al. 2006; Lyvén et al. 2003). An example of a proposed model structure of SRFA was reported by Topping et al. (2005) and can be found in Fig. S1 in the Electronic Supplementary Material (ESM). The mean log K values for proton binding of SRFA are 3.76 and 9.84, respectively (Ritchie and Perdue 2003). The overall charge distribution as a function of pH is presented in the ESM.

All chemicals were dissolved in deionized water (MilliQ Millipore, resistivity = 18.2 MΩ cm) to the target concentrations under stirring for 12 h, and pH was adjusted by adding precise volumes of standardized HCl. The pH electrode (Microelectrode 6.0234.100, Metrohm Nordic) was calibrated using standardized buffer solutions (Thermo Scientific).

TiO_2_ has photocatalytic properties, which may influence the aggregation behavior. All experiments in this work were made under ambient conditions. In a parallel study (unpublished data), batch experiments were performed in order to study the adsorption of 2,3-DHBA on TiO_2_ nanoparticles in both daylight and darkness. No difference in the amount of ligand adsorbed was observed in the two sets of experiments; and therefore, all experiments in the present study were performed in a normal laboratory environment but in the absence of direct daylight. Further, in natural environments, nanoparticle stability may also be affected by the presence of bacteria, although such biotic influences were not investigated here.

### Dynamic light scattering and ζ-potential

The DLS measurements were performed using a Malvern Zetasizer Nano ZS (ZEN 3600, Malvern Zetasizer, Malvern instruments Ltd. with a laser beam of λ = 633 nm and fixed angle at *θ* = 173° at 20 °C). The z-average hydrodynamic diameter (d_H_) measured in DLS refers to the way the particles in the dispersion diffuse within a fluid and was calculated using the Stokes-Einstein equation. The ζ-potential of the particles was obtained from the measured electrophoretic mobility according to Henry’s equation. Theory and equations can be found in the ESM.

### Aggregation experiments

Aggregation studies were focused on investigating the influence of the type and concentration of organic molecules present in suspension over time. Effects of organic molecules on the stability and aggregation rates of TiO_2_ nanoparticles were investigated by time-resolved DLS by monitoring the z-average hydrodynamic diameter (referred to as d_H_, below). Aggregation of particles due to pH changes was avoided by performing the experiments at pH 2.8, since this was the pH of the stable TiO_2_ suspension obtained during synthesis. Aggregation experiments were carried out using DLS with standard operational procedures (SOP) at a fixed measurement position and attenuator that was optimized for each sample. To optimize the measurements and find the detection limit during environmentally relevant conditions, a test at four particle concentrations (10, 20, 50, and 100 mg/L) was performed. The optimal concentration for measuring aggregation rates was 100 mg/L TiO_2_ in order to achieve clear signals with the DLS instrument. A sample preparation was performed according to the following procedure: (1) MilliQ water was adjusted to pH 2.8 using standardized HCl and then mixed with precise volumes of stock solution of the respective organic molecule at pH 2.8, and (2) precise volumes of stock suspension of TiO_2_ nanoparticles were added to the solution. No buffers were used in order to avoid interference with the adsorbing molecules. Suspensions were thoroughly mixed by vortex for 5 s and placed in the instrument before the first measurement point was collected (after 25 s). Each sample was measured every 5 s during 15 min. The change in hydrodynamic diameter over time was followed with DLS after addition of 2,3-DHBA (0.05–3.3 mM), 1,2,4-BTCA (10–70 μM), phthalic acid (0.8–9 mM), and SRFA (0.9–36.4 μM), respectively. A wide range of ligand concentrations was tested in each case and the concentrations reported here were the ones showing noticeable aggregation in the respective system. Particle concentration was 104 mg/L. pH was measured before each DLS measurement and pH was stable at 2.8 (±0.05) during all experiments. An ionic strength of 10 mM was obtained from conductivity measurements compared to that in NaCl solutions and consistent with the expected amounts of residual Cl from the synthesis. The temperature was kept constant at 20 °C in order to avoid changes in viscosity of the medium.

**Fig. 1:**
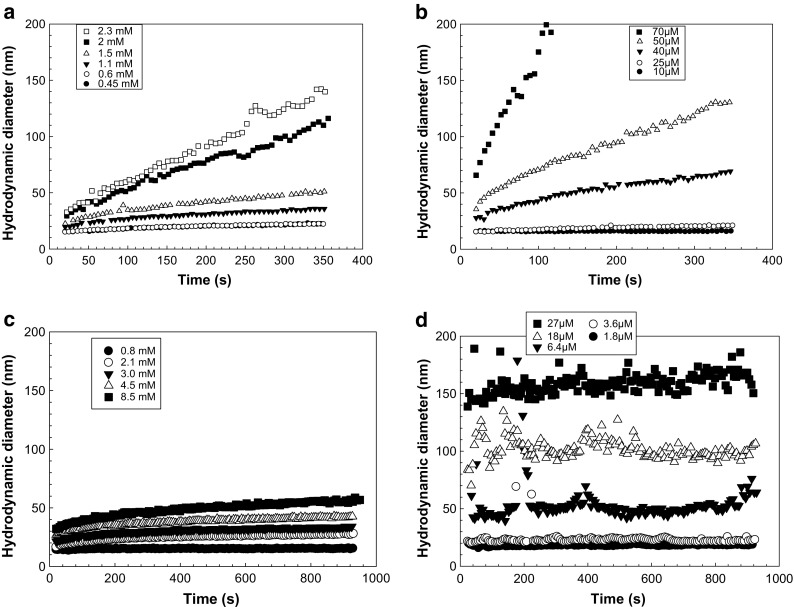
Hydrodynamic diameter after addition of **a** 2,3-dihydroxybenzoic acid (2,3-DHBA), **b** 1,2,4-benzenetricarboxylic acid (1,2,4-BTCA), **c** phthalic acid, and **d** Suwannee river fulvic acid (SRFA), respectively, as a function of time followed by DLS. *Symbols* represent concentrations of the respective organic molecule

**Fig. 2:**
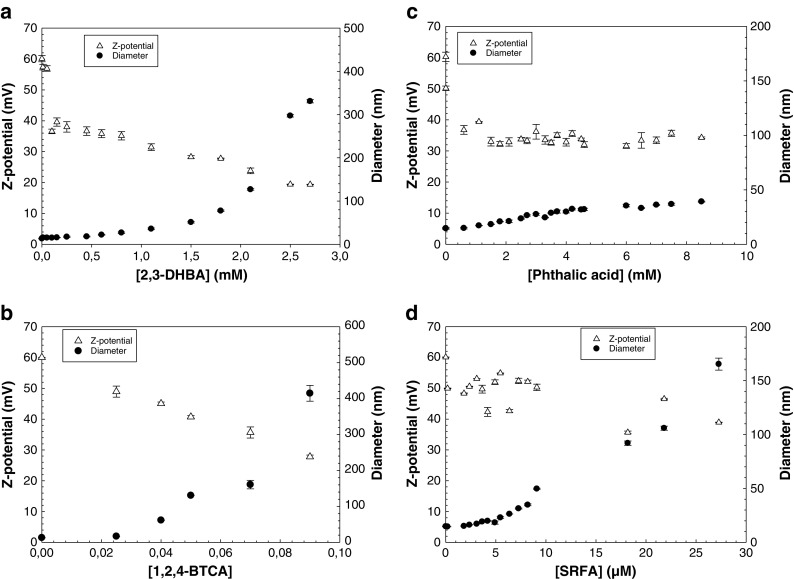
ζ-potential and hydrodynamic diameter of TiO_2_ particles in the presence of **a** 2,3-dihydroxybenzoic acid (2,3-DHBA), **b** 1,2,4-benzenetricarboxylic acid (1,2,4-BTCA), **c** phthalic acid, and **d** Suwannee river fulvic acid (SFRA), respectively. Suspension pH was 2.8 ± 0.05. Particle concentration was 104 mg/L. Please note the different scales. *Error bars* correspond to the standard deviation of three replicates

ζ-potential measurements were determined by DLS following a similar procedure as explained above. However, each sample was measured in three replicates with ten sub runs per measurement. The first data point was achieved after 50 s, the second after 80 s, and the third after 110 s, and the average value was reported for each sample. Corrections due to the influence of d_H_ and ionic strength were done using Oshima’s approximation (Eq. (4) in ESM). The standard error of the experimental data carried out with DLS is ±1.5 mV for ζ-potential measurements and below 5% for z-average diameter measurements in Figs. 1 and 2.

### Batch adsorption experiments

To better understand the mechanisms behind the aggregation behavior of TiO_2_ nanoparticles in the presence of organic molecules, batch samples with a solid concentration of 104 mg/L and total concentrations of the organic molecules ranging from 0.05 to 3.3 mM (2,3-DHBA), 0.05–0.5 mM (1,2,4-BTCA), and 0.8 to 9 mM (phthalic acid), respectively, were prepared. Concentrations were chosen to be the same as the concentrations used in the aggregation experiments. pH was kept constant at 2.8 by initial addition of HCl to all solutions before mixing (the same mixing procedure as in the previous aggregation study) and measured using a combination glass electrode (Metrohm Microelectrode 6.0234.100) that was calibrated in standardized buffers (Reagecon). Samples (30 mL in 50-mL polypropylene test tubes (Falcon)) were kept on a rotator in darkness (Stuart rotator SB3, Bibby Scientific, UK) at 18 rpm, during 10 min for measurements per day, and 0.4 mL of each sample per sampling day was then transferred to centrifugation tubes. Samples were ultracentrifuged for 45 min at a relative centrifugal force (RCF) of 108,900 g (Optima L-90 K Ultracentrifuge, Beckman Coulter) and the concentration of the respective organic molecule in the supernatant was measured with a Cary 4000 UV-Vis spectrophotometer using a quartz cuvette with a 1 cm pathlength at *λ* = 315 nm (2,3-DHBA), *λ* = 276 nm (phthalic acid), *λ* = 290 nm (1,2,4-BTCA), and *λ* = 235 nm (SRFA). Concentrations of ligands remaining in solution were calculated using the Beer-Lambert law, assuming that removal of the molecule from suspension was due to adsorption to the particle surface. The standard error of the adsorption experimental data might be a maximum of 0.05 μmol/m^2^, based on the reproducibility between triplicate batch runs, as well as the stability in the UV-Vis spectroscopic measurements and the results are presented with error bars in Fig. 4.

### Long-term study

A long-term study of the aggregation of TiO_2_ nanoparticles in the presence of 2,3-DHBA and SRFA was performed by monitoring hydrodynamic diameter, pH, and ζ-potential during a time period of 9 months at 20 ± 1 °C. Starting pH was 2.8 and pH was monitored before each DLS measurement throughout the experiment. Test tubes were washed with MilliQ water and HCl to prevent interference with dust particles. TiO_2_ particles with an initial particle diameter of 13 nm were added to solutions of 2,3-DHBA (0.05–3.3 mM) and SRFA (0.9–10.9 mM), respectively, at pH 2.8 in 15-mL polypropylene test tubes (Falcon), and the particle concentration was 104 mg/L. Ligand concentrations were similar to concentrations in the previous aggregation experiment. Samples were kept on the rotator under ambient light at 18 rpm to ensure proper end-over-end mixing during the duration of the experiment. Each sample was measured in three replicates with ten sub runs per measurement. Standard d_H_ and ζ-potential measurements in DLS were performed every second day during the first week and thereafter once a month. The standard errors of the experimental data carried out with DLS was ±1.5 mV for ζ-potential measurements and below 5% for z-average diameter measurements in Figs. 5 and 6. In Fig. 6b, the standard errors are below 10% for the experimental data of ligand (SRFA) concentration >40 μM tested (where the z-average diameter is in the micrometer range).

### High-resolution transmission electron microscopy

Samples for TEM analysis in the present work were prepared by placing a drop of the nanoparticle fluid on a holey carbon-coated Cu grid. The grid was put on a piece of filter paper to immediately remove any excess liquid, thereby reducing drying artifacts like particle agglomeration. High-resolution TEM analysis was carried out using a FEI Titan 80-300 operating at 300 kV. All high-resolution images acquired were analyzed with the Digital Micrograph software.

## Results and discussion

### Aggregation of TiO_2_ nanoparticles

The colloidal stability of TiO_2_ in the presence of organic molecules was investigated by simultaneously monitoring the changes in d_H_ and ζ-potential during the reactions. Since our synthesized TiO_2_ nanoparticles are stable in suspension at pH 2.8, the first effort to fundamentally investigate the aggregation behavior of TiO_2_ in the presence of organic molecules was performed at pH 2.8. Results showed that the d_H_ change in time was dependent on the concentration of the organic molecule added in all systems investigated and increased faster at higher ligand-to-solid ratios (Fig. 1). Both 2,3-DHBA (Fig. 1a) and 1,2,4-BTCA (Fig. 1b) gave rise to a higher increase in d_H_ with time, than did phthalic acid (Fig. 1c). SRFA (Fig. 1d) showed fast aggregation immediately after mixing but a stable d_H_ after a few seconds. 1,2,4-BTCA was found to be the most effective destabilizing ligand in terms of the mass ratio between the organic molecule and nanoparticle. Specifically, the highest concentration of investigated 2,3-DHBA (2.3 mM) resulted in a d_H_ of 140 nm in 350 s (Fig. 1a). During the same time period, only 50 μM of 1,2,4-BTCA was required in order to reach the same d_H_ (Fig. 1b), which indicates a faster aggregation in the latter case. On the other hand, aggregation in the presence of phthalic acid (Fig. 1c) was much slower even at very high concentrations (8.5 mM). This suggested weak or no interactions between phthalic acid and TiO_2_, which was confirmed with the batch adsorption experiments (described hereafter) where no adsorption of phthalic acid to the particle surface was observed after 10 min (Fig. 4b).

These results indicate that different types and orientations of functional groups strongly affect the aggregation behavior of TiO_2_ nanoparticles by interacting differently with the particle surfaces. The order of induced aggregation follows the same order as the overall charge density (Fig. S1 in ESM), except for SRFA, which presents a plateau: phthalic acid <2,3-DHBA <1,2,4-BTCA. Therefore, in these cases, the aggregation might be induced by compression of the double layer (as it is the case for multivalent cations).

Fulvic acids are relatively large compounds with a high extent of functional groups, and the initial aggregation during the first 25 s in the TiO_2_-SRFA system was relatively fast (Fig. 1d). The TiO_2_-SRFA system reached a semi-stable condition where the hydrodynamic diameter was dependent on the total concentration of SRFA. This result indicates that SRFA increased the electrostatic repulsion, possibly by providing electrosteric stabilization after adsorption to the particle surface. This is also visualized in Fig. 2d, where the ζ-potential was highly positive for all samples. This behavior might be due to the large size and the high charge density (Fig. S2 in ESM) of SRFA, which enables it to cover the total surface of the particle to a higher extent and induce electrosteric stabilization. The differences observed between SRFA and the model compounds are expected to be due to the large size, as well as the presence of several functional groups on the SRFA molecule. Besides electrostatic and specific interactions between functional groups and the particle surface sites, adsorption of NOM molecules such as SRFA may also be dependent on hydrophobic interactions because of the macromolecular nature of NOM molecules; a feature that might not be as pronounced for the smaller model molecules.

Additionally, data for the first 25–60 s were plotted and presented in Fig. S5 as the slopes of the initial aggregation for all the molecules vs log concentrations compared with values for particles in NaCl at a concentration of 100 mg/L. The results show how efficient the organic molecules are in destabilizing the particles, i.e., a significantly larger NaCl concentration would be required to generate the same destabilizing effect as the organic molecules.

Figure 2 shows the ζ-potential, as well as hydrodynamic diameter as a function of ligand concentration at pH 2.8. Data were collected 25 (±3) seconds after the addition of TiO_2_ nanoparticles to the respective ligand solution. Generally, ζ-potentials decreased while particle diameters increased with increased concentration of the organic molecule in all systems, although some variations were found. In the 2,3-DHBA-TiO_2_ system (Fig. 2a), the ζ-potential dropped rapidly from 60 to 40 mV as the ligand concentration changed from 0 to 0.1 mM. This was followed by a decrease from approx. 40 to 20 mV as the ligand concentration increased from 0.1 to 3.3 mM. During the same experiment, initial growth of particles was small and the particle diameter remained below 100 nm up to approx. 2 mM of 2,3-DHBA. At the highest concentrations of 2,3-DHBA, particles grew significantly to more than 300 nm in diameter. At pH 2.8, 2,3-DHBA is close to its p*K*a_1_, thus carrying some negative charge on the carboxyl group, which allows electrostatic attraction to the positively charged TiO_2_ surface. Further, the phenolic hydroxyl groups on 2,3-DHBA are assumed to take part in the surface complexation through hydrogen bonding, as was shown previously to be the case for catechol on TiO_2_ (anatase) surfaces (Gulley-Stahl et al. 2010; Lana-Villarreal et al. 2005), as well as DOPA on TiO_2_ (rutile) surfaces (Bahri et al. 2011). Formation of 2,3-DHBA-TiO_2_ surface complexes results in neutralization of the surface charge, which leads to aggregation strongly dependent on the ligand-to-solid ratio.

The same phenomenon occurs in the case of 1,2,4-BTCA, although neutralization is more effective due to the presence of three carboxyl groups that are deprotonated at relatively low pH (Table 1). At 1,2,4-BTCA concentrations above 0.05 mM, particles grew larger than 100 nm and the particle size increased dramatically thereafter (Fig. 2b). At 0.09 mM of 1,2,4-BTCA, particles were larger than 400 nm in diameter, which indicates very strong interactions between the ligand and the particle surface affecting the aggregation rate. The decrease in colloidal stability was also demonstrated by the drop in the ζ-potential from 60 to 25 mV with increased ligand concentration from 0 to 0.1 mM. At pH 2.8, a significant number of 1,2,4-BTCA molecules carry negative charges that can interact with the surface and compress the electrical double layer. Further, the position of the carboxyl groups of 1,2,4-BTCA might enable bridging between particles, which will in turn enhance the aggregation.

In the case of phthalic acid (Fig. 2c), large increases in ligand concentration up to 8 mM gave rise to only small changes in hydrodynamic diameter (from 20 to 40 nm). In parallel, after a drop from 60 to 40 mV after the first ligand addition, the ζ-potential remained more or less constant at 30–40 mV throughout the experiment. This indicates very weak (if any) interactions between the ligand and the surface, and phthalic acid does not seem to significantly affect the colloidal stability of TiO_2_ nanoparticles at this pH. The lowest p*K*a value for phthalic acid is 2.87 (Table 1), which means that at pH 2.8 the overall charge density is very low (see Fig. S1 in ESM) and the molecules are not electrostatically attracted to the positively charged TiO_2_ surface to a high extent.

In the presence of SRFA (Fig. 2d), particle sizes were relatively stable at low concentrations (<9.1 μM) of SRFA. As the concentration of SRFA was increased to 18.1 μM and above, larger aggregates (>100 nm) started to form and the ζ-potential decreased (<30 mV), which indicated a less stable system. The mechanism by which SRFA adsorbed to the surface is expected to be a combination of hydrophobic interactions and bidentate complex formation of surface sites with functional groups on SRFA. There is also the possibility of multilayer adsorption of SRFA at the TiO_2_ surface (Kleber et al. 2007; Tiller and O’Melia 1993). The decrease in ζ-potential to negative values that was found in the previous study of von der Kammer et al. (2010) was not obtained here, which is thought to be due to lower concentrations of SRFA and the lower pH in this study. An increase in SRFA concentration above the maximum concentration used in this work might result in a reestablished stabilization of the particles, hence decreasing aggregation rate due to electrostatic repulsion between negatively charged surfaces as a result of SRFA adsorption. A comparison of the general trends in Fig. 2 suggested that 1,2,4-BTCA was the model molecule that best described the behavior of SRFA in terms of changes in ζ-potential and TiO_2_ particle size at low ligand concentrations. This might be explained by the molecular structure of 1,2,4-BTCA and the distribution of carboxyl groups that can bridge between TiO_2_ particles, which is a plausible mechanism also in the case of SRFA.

The fact that the ζ-potential remained positive throughout all the experiments in this study indicates that the dominant charge of the aggregates at this pH is still dominated by the strong positive surface charge of the TiO_2_ particles. This charge, however, was not sufficient to create stable colloidal aggregates except for the case of SRFA, as seen in Fig. 1d.

### TEM analysis

**Fig. 3:**
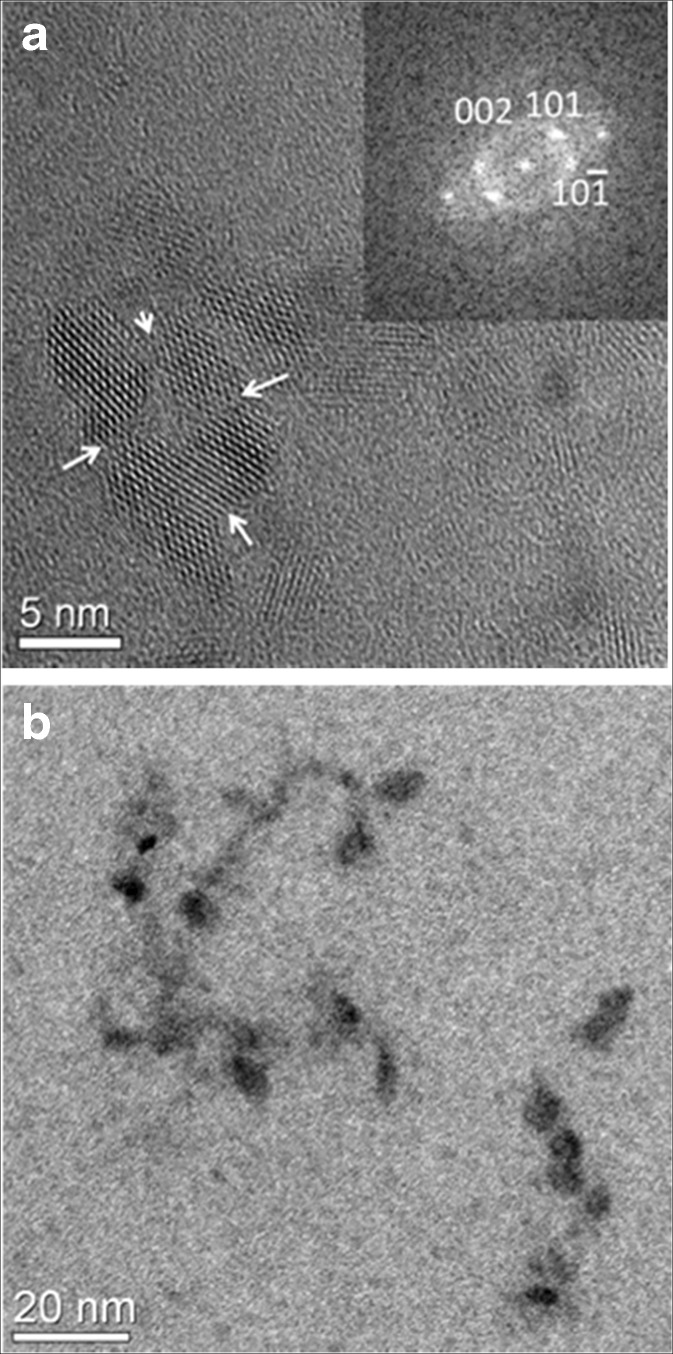
High-resolution TEM image of a cluster of TiO_2_ (anatase) nanoparticles interacting with **a** phthalic acid (1.1 mM in solution) and **b** 2,3-DHBA (1.1 mM in solution). The interface region between the particles is marked by a *neck* and visualized with *arrows* in **a**. The *inset* shows the diffraction pattern obtained from a fast Fourier transform. *Scale bars* represent **a** 5 and **b** 20 nm

High-resolution TEM analysis of TiO_2_ (anatase) nanoparticles in the presence of phthalic acid and 2,3-DHBA, respectively, showed that the typical primary particle diameter was around 3–5 nm (Fig. 3). Figure 3a shows a cluster of anatase particles attached along the {001} and {110} planes after interaction with phthalic acid (1.1 mM in solution). The interface region is marked by a neck (arrowed in the image), showing that the cluster indeed comprised several particles. Furthermore, a tendency for particle attachment along certain crystallographic planes, so called oriented attachment, was observed in many particle aggregates. The sizes of the aggregates, similar to that shown in Fig. 3a, are in agreement with DLS measurements of the same sample, where the hydrodynamic diameter in solution was measured to be 20 nm (Fig. 1c).

Figure 3b shows anatase nanoparticles that have interacted with 2,3-DHBA (1.1 mM in suspension). In the presence of 2,3-DHBA, the particles tend to form chain-like aggregates. However, sample preparation is important to take into account when comparing results from TEM and DLS since TEM is measured on a dried sample and cannot be directly compared to aggregate structures in suspension.

### Batch adsorption experiments

**Fig. 4:**
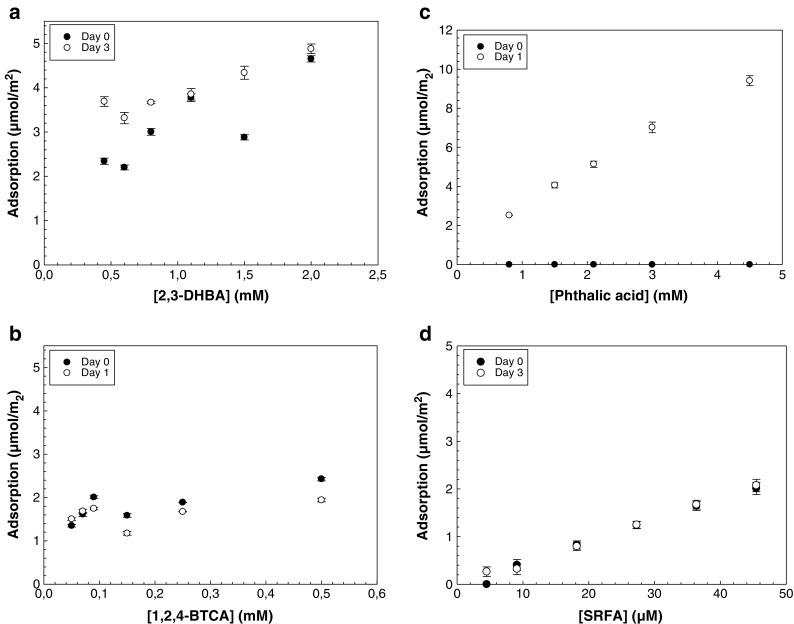
Adsorption of **a** 2,3-DHBA, **b** 1,2,4-BTCA, **c** phthalic acid, and **d** SRFA on TiO_2_ nanoparticles displayed as adsorption (μmol/m^2^) as a function of the initial total concentration of the respective organic molecule. Initial particle size = 13 nm; BET surface area = 206 m^2^/g; solid conc = 104 mg/L. *Error bars* correspond to standard deviation of three replicates

**Fig. 5:**
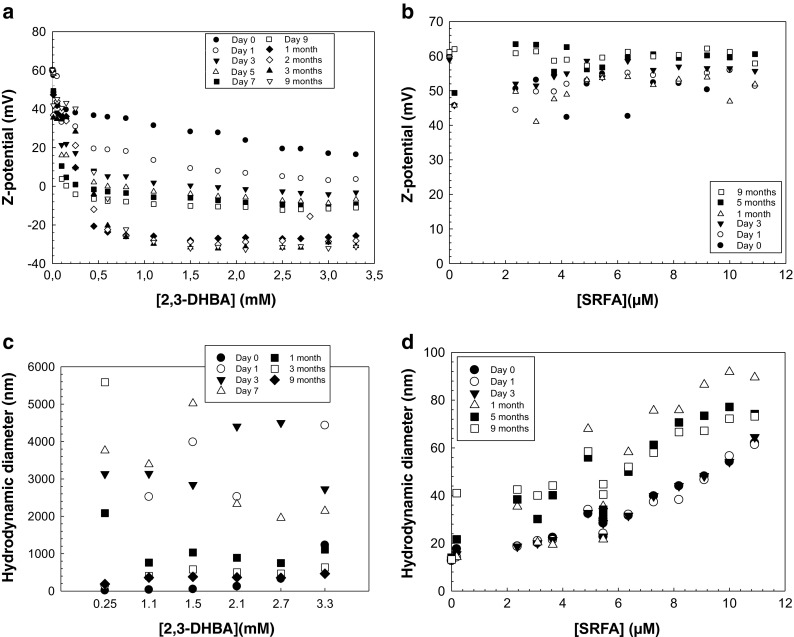
ζ-potential and hydrodynamic diameter of TiO_2_ nanoparticles over time and at different total concentrations of **a**, **c** 2,3-dihydroxybenzoic acid (2,3-DHBA) and **b**, **d** Suwannee river fulvic acid (SRFA). pH was constant at 2.80 ± 0.05. Particle concentration was 104 mg/L. *Symbols* represent time that passed since the start of the experiment

Figure 4a shows the adsorption of 2,3-DHBA on the TiO_2_ surface, at day 0 (approx. 10 min after mixing) and day 3 of the experiment. At the lowest ligand concentrations (0.5 mM 2,3-DHBA), the amount of adsorption observed was around 2 μmol/m^2^ at day 0. As the ligand concentration increased to 2 mM, a maximum of 4.6 μmol/m^2^ was adsorbed, which corresponds to 5% adsorption. After 72 h of equilibration (day 3), the amount of 2,3-DHBA adsorbed increased to approx. 3.3 μmol/m^2^ at the lowest ligand concentrations, but remained more or less constant (4.6 μmol/m^2^) at the highest ligand concentration. This indicates that time influences the interactions between TiO_2_ nanoparticles and 2,3-DHBA, especially at the lowest ligand concentrations used in this study, and a further study on this phenomena is shown in Fig. 5.

No significant effect of neither ligand concentration nor time was visible in the adsorption of 1,2,4-BTCA on TiO_2_ nanoparticles (Fig. 4b). On the other hand, the ligand concentration range tested was rather narrow (0.05–0.5 mM of 1,2,4-BTCA) compared to that of the other experiments in Fig. 4, and it is possible that larger amounts of 1,2,4-BTCA would need to be added to the dispersion in order to see clearer trends. The concentrations of 1,2,4-BTCA investigated here were selected in order to be comparable with the aggregation data (Figs. 1b and 2b). In this concentration range, the adsorption was about 1.5–2 μmol/m^2^ after 10 min on the rotator (Fig. 4b). Changes in adsorption after 24 h compared to those after 10 min were negligible, which indicates that the system reached equilibrium faster compared to the other molecules investigated. The aggregation study indicated no or very little interaction between phthalic acid and the TiO_2_ particle surfaces during the first 15 min after mixing (Figs. 1c and 2c), and this was confirmed in the adsorption experiments. Figure 4c shows that no phthalic acid was adsorbed after 10 min on the rotator (day 0). However, after equilibration for 24 h, there was a clear trend of increased amount of adsorption as the ligand concentration increased. Additional DLS and ζ-potential measurements were performed, and after 24 h it showed an increase in size and decrease in ζ-potential (Fig. S3) compared to measurements during the first 15 min (Figs. 1 and 2).

Increase in total concentration of SRFA gave rise to increased amount of adsorption (Fig. 4d) but no significant difference in adsorption after 10 min on the rotator (day 0) compared to day 3 was visible in the experiments. This indicates that the adsorption of SRFA takes place during a matter of minutes or hours rather than days.

The adsorption of all four molecules investigated, 2,3-DHBA, 1,2,4-BTCA, phthalic acid, and SRFA, was shown to be dependent upon ligand concentration, which is in agreement with the that of the aggregation study (Figs. 1 and 2) and that of the earlier studies in this field (Pettibone et al. 2008).

### Effect of solid concentration

To investigate whether particle concentration affects the aggregation behavior, precise amounts of TiO_2_ nanoparticles were added to 2,3-DHBA solutions (0.25–2.3 mM) to obtain two particle concentrations (104 and 363 mg/L, respectively), and the results are shown in Fig. S4 in the ESM. DLS measurements showed that higher particle concentration (363 mg/L) resulted in faster aggregation rates, and larger aggregates were formed with time (Fig. S4b) compared to those in a less concentrated (104 mg/L) suspension (Fig. S4a). For example, in the presence of 1.1 mM 2,3-DHBA, a small increase in diameter from 20 to 35 nm was recorded over a time period of 350 s at the lower particle concentration (Fig. S4a). However, much larger aggregates (up to 235 nm in diameter) were formed during the same time period at the higher particle concentration (Fig. S4b). This is consistent with DLVO theory, which predicts an increased number of collisions as the number of particles increases (Arvidsson et al. 2011). In order to interpret the effect of particle concentration on the aggregation quantitatively, it is necessary to also take the ligand-to-solid ratio into account since this affects the attachment efficiency. However, the trends in Fig. S4 clearly indicate that solid concentration is important for the colloidal stability.

### Long-term study

In order to investigate long-term effects on the colloidal stability of TiO_2_ nanoparticles in the presence of 2,3-DHBA and SRFA, respectively, ζ-potential and d_H_ were measured regularly during a time period of 0–9 months (Fig. 5). During the first month, results showed that at concentrations higher than 0.1 mM 2,3-DHBA, ζ-potentials decreased significantly with time and after 3 days surface charge neutralization occurred at 1.5 mM 2,3-DHBA (Fig. 5a). At higher concentrations of 2,3-DHBA (1.8–3.3 mM), the particles obtained a negative ζ-potential that decreased further with time. After 1 month, the system started to stabilize and the ζ-potential decreased slightly from −25 to <−30 mV during the last months of the experiment. The observed behavior at different ligand concentrations might be due to varying levels of surface coverage. At pH 2.8, the TiO_2_ surface is positively charged, a charge that is progressively being neutralized as 2,3-DHBA molecules adsorb. Both the carboxyl group and the hydroxyl groups are thought to be involved in the binding of 2,3-DHBA to surface sites, possibly through outer-sphere and/or hydrogen bonding (Bahri et al. 2011; Gulley-Stahl et al. 2010). At low concentrations of 2,3-DHBA, the surface is most likely partly covered followed by full coverage and possibly even stacking of molecules at the surface at higher ligand concentrations. The involvement of hydroxyl groups, as well as stacking of molecules, could mean that negative carboxyl groups point away from the surface, which results in a net negative surface potential. Fourier transform infrared spectroscopic (FTIR) measurements that reveal the character of the bonds between 2,3-DHBA and the surface of TiO_2_ nanoparticles are currently being undertaken and will be presented in a forthcoming publication. Initial adsorption of ligands to the surface may be very fast, followed by a slower rearrangement of surface complexes as the system tries to reach equilibrium. This rearrangement will alter the surface potential, which in turn affects the aggregation behavior.

Interactions of SRFA with TiO_2_ nanoparticles over a time period of 9 months did not change ζ-potentials significantly compared to thoseof bare particles in the chosen SRFA concentration range as the ζ-potentials remained strongly positive (50–60 mV) for all samples investigated (Fig. 5b). This indicates that no or little interaction between SRFA and TiO_2_ surface occurs which might be due to a low concentration of SRFA in this experiment.

**Fig. 6:**
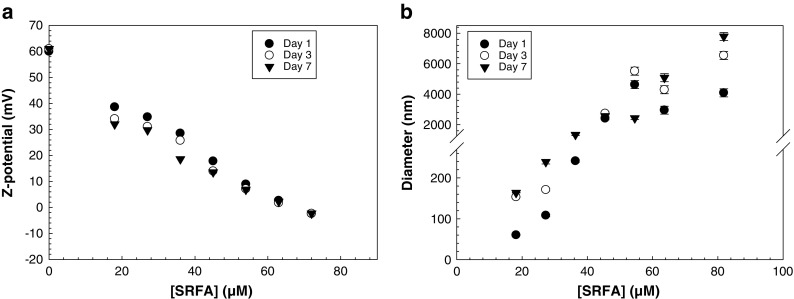
ζ-potential of TiO_2_ nanoparticles over time and at different total concentrations of Suwannee River fulvic acid (SRFA). pH was constant at 2.80 ± 0.05. Particle concentration was 104 mg/L. *Symbols* represent time that passed since the start of the experiment. *Error bars* correspond to the standard deviation of two replicates

The hydrodynamic diameter was measured for all samples and shown in Fig. 5c and d, and the data can be found in the ESM (Table S1 and S2). In the 2,3-DHBA-TiO_2_ system (Fig. 5a and Table S1a) and at ligand concentrations of 0.1 mM and above, aggregate diameters increased up to several microns during the first month. This was followed by a decrease during the following months of the experiment, and after 9 months most aggregates had a particle diameter of only a few hundred nanometers. The disaggregation may indicate the presence of loose aggregates that may disassociate as the system is equilibrating as a result of rearrangement of surface complexes. The same result was not found in the case of SRFA (Fig. 5d and Table S2a). Instead, the presence of SRFA gave rise to only slight increases in particle sizes, and after 9 months, most particles had a hydrodynamic diameter between 30 and 70 nm. The adsorption data in Fig. 4 confirms that there is no or very little interaction between SRFA and the particle surface at these low concentrations (<10 μM SRFA). This was also observed in a recent study of TiO_2_ nanoparticles with humic acid, where no large variations in size or ζ-potentials were observed during a time period of 12 h (Lee et al. 2016). Since no significant effect on neither ζ-potential nor hydrodynamic diameter was observed in Fig. 5b and d, an additional experiment was performed at which higher concentrations of SRFA (18–82 μM) were used (Fig. 6a and b). The experiment proceeded during 1 week and both ζ-potential and size were recorded at day 1, day 3, and day 7. The ζ-potential decreased with the increased concentration of SRFA, and at around 68 μM, the surface was neutralized (Fig. 6a). At the highest SRFA concentration, the ζ-potential was negative. At the lowest concentrations of SRFA (i.e. <35 μM), there is a small trend towards a lower ζ-potential during the first 7 days; however, this trend is not evident (statistically significant) for the larger concentrations. The aggregate sizes increased with an increased concentration of SRFA. At the three lowest concentrations, the particle sizes remained below 250 nm during the first 3 days (Fig. 6b). However, after the addition of 45 μM SRFA, the size increased significantly to 2.5 μm and increased further with concentration. This is in correlation with the ζ-potential measurements of the same samples. At 45 μM, the ζ-potential was below 20 mV, indicating destabilization of the system.

The results in Fig. 6 can be further explained by the adsorption data of the same system (Fig. 4d), showing that an increased concentration of SRFA leads to an increased amount adsorbed to the surface, which in turn results in destabilization of the system.

In order to extrapolate results to more environmentally relevant conditions, it would be beneficial to repeat the experiments at near-neutral pH, and this will be done in a subsequent step of this research. Increasing the pH is challenging since our synthesized TiO_2_ nanoparticles have pH_PZC_ near pH 6 and aggregation will occur even before the addition of organic molecules, which would add to the complexity of the system as the number of variables increases. Increased pH would likely result in a different aggregation behavior, since both the surface potential of TiO_2_ and charges of functional groups on organic ligands depend strongly on pH. The amount of negative charge at the TiO_2_ surface increases with pH, which is also the case for the organic ligands depending on their respective p*K*a (Table 1). A small increase of only a few pH units from 2.8 up to the pH_PZC_ might result in a higher tendency of the ligands to interact electrostatically with the surface, since the surface still has an excess of positive charge that will attract negatively charged ligands. SRFA has a relatively low p*K*a (3.76), which means that free functional groups (situated on surface complexed SRFA) that are not directly bound to the TiO_2_ surface might provide the surface with a net negative charge at near-neutral pH. The question remains whether the presence of organic ligands in suspension resulting in surface complexation or the pH increase itself will have the greatest impact on the formation of larger aggregates. Above the pH_PZC_, interactions expected to take place, if any, might be hydrogen bonding of ligands to the surface through the phenolic OH groups.

## Conclusion

This study presents evidence on the importance of studying processes at the molecular level in order to understand the mechanisms behind nanoparticle stability. The use of small organic model molecules demonstrated the significance of the type and position of the functional groups for both the colloidal stability and the adsorption of ligands, since their interactions with the particle surface are, to a large extent, driven by electrostatic forces. Ionic strength, temperature, and pH were kept constant in the present study and this was the first step to fundamentally investigate the effects of ligand-to-solid ratio and type of organic molecule on the colloidal stability of synthesized TiO_2_ nanoparticles (anatase) in both short- and long-term perspectives. Our results show that nanoparticles interact with organic molecules depending on particle concentration, ligand concentration, type and/or position of functional groups, and time in suspension.

The impact of nanoparticles on organisms living in the water or sediment is highly dependent on the colloidal stability, since this will affect the exposure of nanoparticles to these ecosystems. There is a need for long-term time studies of similar systems to be able to get a better understanding of the fate and behavior of synthetic nanomaterials and thereby reduce uncertainty in nanomaterial risk assessments.

## Electronic supplementary material

ESM 1 (DOCX 2810 kb)

Electronic supplementary material includes the proposed structure of SRFA; theory behind DLS measurements; data from the long-term study of the aggregation of TiO2 nanoparticles in the presence of 2,3-DHBA and SRFA, respectively (Fig. 5); theory and figures showing the overall charge density variation as a function of pH for 2,3-DHBA, SRFA, phthalic acid, and 1,2,4-BTCA; figures showing the ζ-potential and hydrodynamic diameter of TiO2 particles in the presence of phthalic acid; figures showing the effect of solid concentration on the hydrodynamic diameter of TiO2 particles; and a figure showing the slope of the aggregation rates during the first 60 s in the aggregation experiments.
