# Supplementary material for: Influence of organic molecules on the aggregation of TiO2 nanoparticles in acidic conditions
Source: J Nanopart Res. 2017 Apr 4;19(4):133. doi: 10.1007/s11051-017-3807-9 (PMC5380707; doi:10.1007/s11051-017-3807-9)
Supplement: Supplementary file 1 — (DOCX 2810 kb) [file 11051_2017_3807_MOESM1_ESM.docx]

**ELECTRONIC SUPPLEMENTARY INFORMATION**

Influence of Organic Molecules on the Aggregation of TiO_2_ Nanoparticles in Acidic Conditions

*Submitted to Journal of Nanoparticle Research*

Karin Danielsson^1,^*, Julián A. Gallego-Urrea^2^, Martin Hassellöv^2^, Stefan Gustafsson^3^, Caroline M. Jonsson^1,^

1. University of Gothenburg, Department of Chemistry and Molecular Biology, 412 96 Gothenburg, Sweden
2. University of Gothenburg, Department of Marine Sciences, 412 96 Gothenburg, Sweden
3. Chalmers University of Technology Department of Applied Physics, 412 96 Gothenburg, Sweden

Corresponding author (marked with *):

Email: karin.danielsson@chem.gu.se

Tel: (+46) 31 786 9092)

**
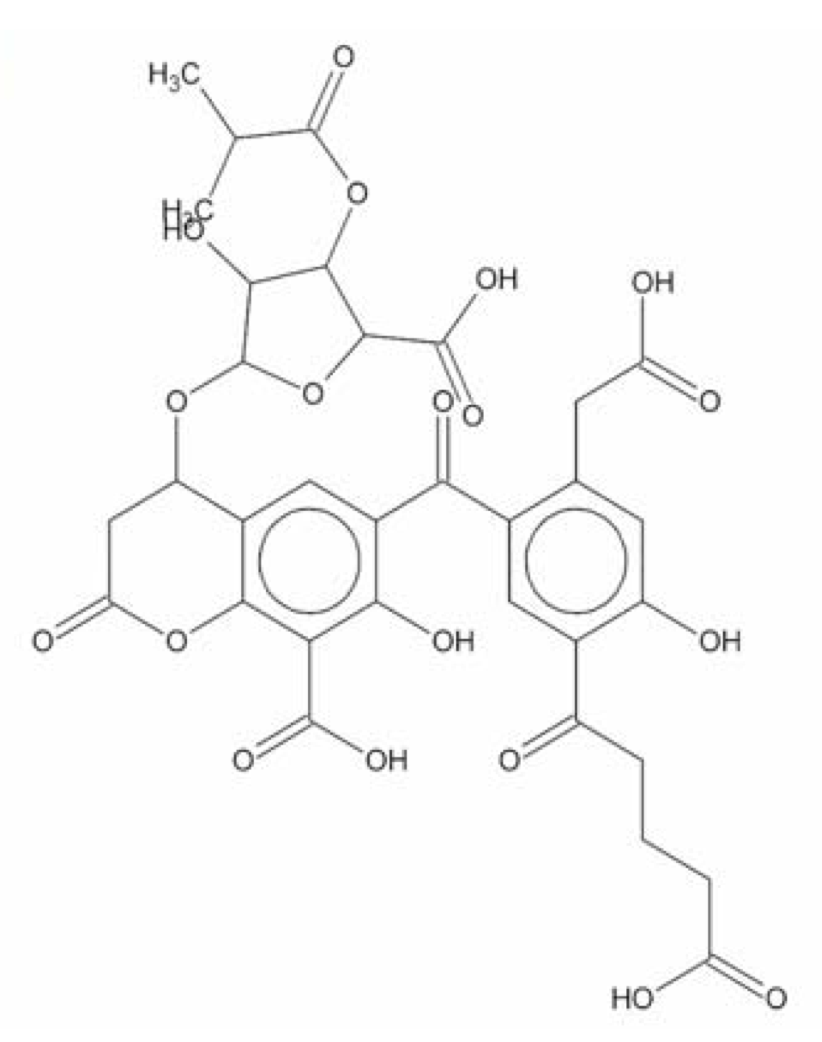
**

Fig. S1 Proposed model structure of Suwannee River Fulvic Acid (SRFA). Structure taken from ([Topping et al. 2005](#_ENREF_5)).

**Dynamic Light Scattering (DLS) and ζ-potential**

The DLS measurements were performed using a Malvern Zetasizer Nano ZS (ZEN 3600, Malvern Zetasizer, Malvern instruments Ltd).In DLS, autocorrelation functions of the fluctuations in scattered light at a fixed angle are obtained, and the exponential decay of the first-order autocorrelation function is related to the ensemble-averaged diffusion coefficient. Using the Stokes–Einstein equation (2), it is possible to find the z-averaged hydrodynamic equivalent diameter (Nobbman and Morfesis 2009).

$d_{H}= \frac{k_{B}\cdot T}{3\cdot\pi\cdot\eta\cdot D}$ (1)

Where d_H_ is the hydrodynamic diameter, k_B_ is the Boltzmann’s constant, T is the temperature, η is the viscosity of the medium and D is the diffusion coefficient.

The electrophoretic mobility of the particles was measured by Zetasizer Nano, and the ζ-potential was obtained from the measured electrophoretic mobility according to the Henry’s equation (eq. 3)

$u= \frac{2\cdot\varepsilon\cdot\varepsilon_{0}\cdot\zeta}{3\cdot\eta}\cdot f(\kappa a)$ (2)

Where u is the electrophoretic mobility, ε is the medium dielectric constant, ε_0_ is the vacuum permittivity, ζ is the zeta potential, κ is the inverse Debye length and *a* is the particle hydrodynamic radius. The inverse Debye length is defined as:

$\kappa=\left( \frac{2000\cdot e^{2}\cdot N_{A}\cdot I}{\epsilon\cdot k_{B}\cdot T} \right)^{\frac{1}{2}}$ (3)

Where e is the elementary charge, N_A_ is the Avogadro’s number, I is the ionic strength defined as I=1/2.∑z_i_^2^.M_i_ with z_i_ and M_i_ the ion i charge and molar concentration, respectively.

Equation 3 was used for calculating ζ-potentials with the correction term f(*κa*) given by the Oshima’s equation (eq. 5) ([Ohshima 1995](#_ENREF_3)).

$f(Ka)=1+ \frac{1}{{2\left[ 1+\left[ \frac{2.5}{Ka\left[ 1+2^{\left[ -Ka \right]} \right]} \right] \right]}^{3}}$ (4)

**Long-term Study**

(Data for Figure 5)

**Table S1a.** z-average hydrodynamic diameter measured with DLS (data for Fig. 5c)

**[TiO_2_] = 104 mg/L pH = 2.8 T = 20°C**

**Table S1b.** Calculated z-potentials using Henrys and Smulokowskis equation. Measurements made with DLS (data for Fig. 5a)

**Table S2a.** z-average hydrodynamic diameter measured with DLS (data for Fig. 5d)

**[TiO_2_] = 104 mg/L pH = 2.8 T = 20°C**

**Table S2b.** Calculated z-potentials using Henrys equation. Measurements made with DLS (data for Fig. 5b)

**Calculation of the overall charge density (extent of dissociation) for a polyprotic weak acid ignoring electrostatic effects.**

The dissociation of a polyprotic weak acid in water follows:

$$H_{3}A\underset{\leftrightarrow}{K_{a1}}H^{+}+H_{2}A^{-}\underset{\leftrightarrow}{K_{a2}}H^{+}+HA^{2-}\underset{\leftrightarrow}{K_{a3}}H^{+}+A^{3-}$$

The average charge density, CD, of the acid in water can be defined in equivalents per mol as a function of pH ([Tipping 2005](#_ENREF_4)):

$$CD=\frac{\left[ H_{2}A^{-} \right]+2\cdot\left[ HA^{2-} \right]+3\cdot\left[ A^{3-} \right]}{\left[ H_{3}A \right]_{0}}$$

The different species concentrations of the acid can be calculated from the Ka values:

$$\left[ H_{2}A^{-} \right]=\frac{K_{a1}\cdot\left[ H_{3}A \right]}{\left[ H^{+} \right]}$$

$$\left[ HA^{2-} \right]=K_{a2}\frac{K_{a1}\cdot\left[ H_{3}A \right]}{\left[ H^{+} \right]^{2}}$$

$$\left[ A^{3-} \right]=K_{a3}\cdot K_{a2}\cdot\frac{K_{a1}\cdot\left[ H_{3}A \right]}{\left[ H^{+} \right]^{3}}$$

The charge balance (neglecting the presence of OH^-^) is defined as:

$$\left[ H^{+} \right]=\left[ H_{2}A^{-} \right]+\left[ HA^{2-} \right]+\left[ A^{3-} \right]$$

And the mass balance for H_3_A:

$$\left[ H_{3}A \right]_{0}=\left[ H_{3}A \right]+\left[ H_{2}A^{-} \right]+\left[ HA^{2-} \right]+\left[ A^{3-} \right]$$

The CD can then be plotted for 2,3-Dihydroxybenzoic acid (2,3-DHBA), phthalic acid and 1,2,4-benzenetricarboxylic acid (trimellitic acid or 1,2,4-BTCA), knowing their respective experimental dissociation constants (Table 1 in main document).

The overall charge per unit of mass for the fulvic acid standard (batch 2S101F) from the International Humic Substances Society (IHSS, University of Minnesota, St. Paul, MN, USA) can be obtained using the modified Henderson-Hassebalch equation and the parameters provided by the IHSS ([International Humic Substances Society 2010](#_ENREF_2)) and a molecular weight of 551 Da ([Fattahi and Solouki 2003](#_ENREF_1)).

Results from all molecules used in this work are presented in figure S2. The ranking from low to high CD at this range of pH is phthalic acid ≈ 2,3-DHBA < 1,2,4-BTCA < SRFA.

| 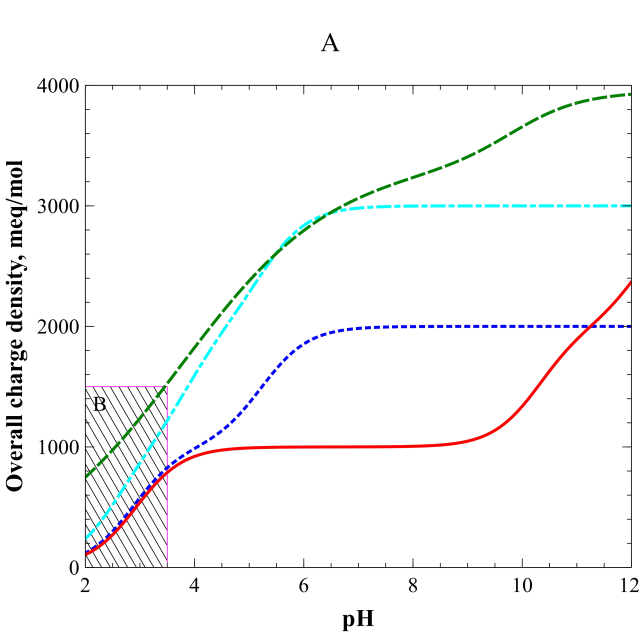 | 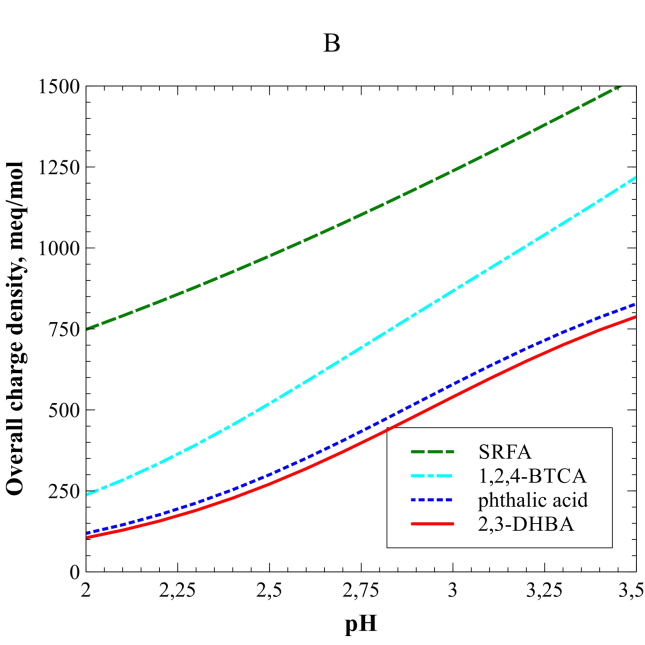 |
| --- | --- |

Fig. S2 Overall charge density variation as a function of pH for 2,3-DHBA, SRFA, phthalic acid and 1,2,4-BTCA. The panel A contains a wide pH range while the panel B is an extract highlighting the pH values used in this work. The shadowed region indicates the region extracted for panel B.

**Fig. S3** ζ-potential (a) and hydrodynamic diameter (b) of TiO_2_ particles in the presence of phthalic acid. pH was constant at 2.80±0.05. Particle concentration was 104 mg/L. Symbols represent time that passed since the start of the experiment.


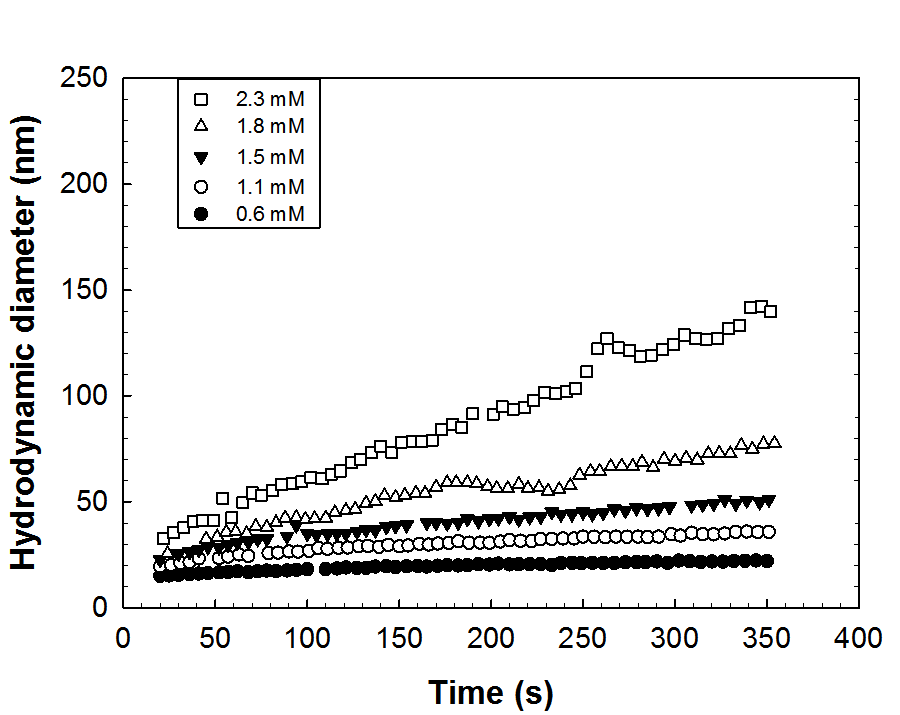

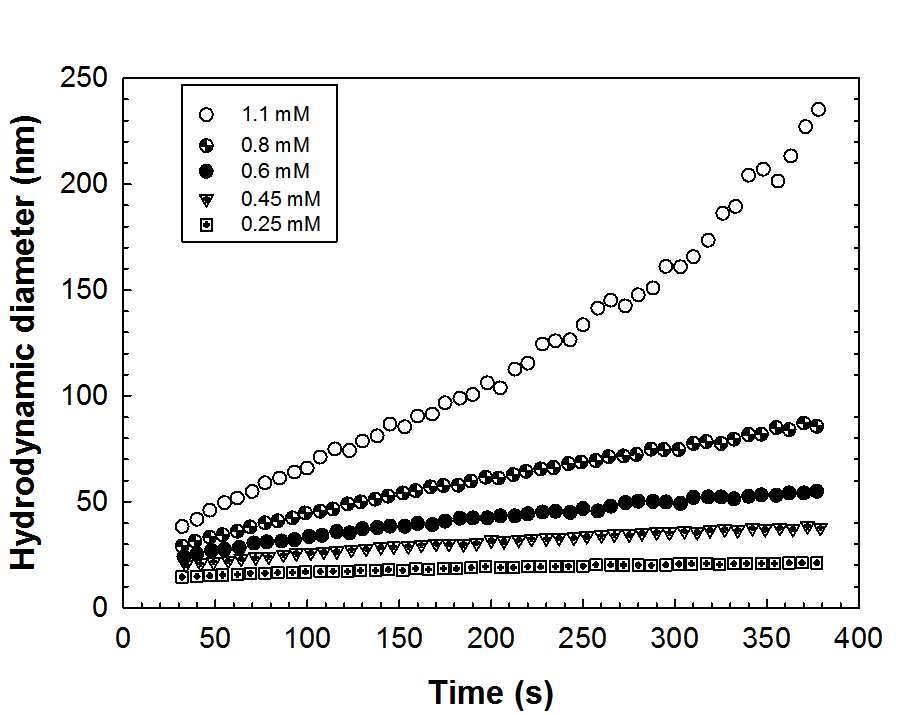


b

a

**Fig. S4**

The effect of solid concentration on the hydrodynamic diameter of TiO_2_ in the presence of 2,3-dihydroxybenzoic acid (2,3-DHBA) and as a function of time at pH 3.0. Solid concentration was a) 104 mg/L, and b) 363 mg/L. Symbols represent different concentrations of 2,3-DHBA


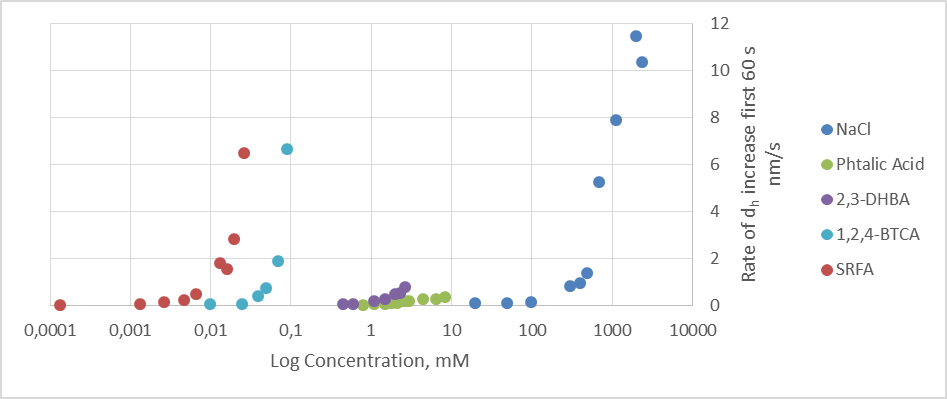


**Fig. S5**

Slope of the aggregation rates of TiO_2_ during the first 25-60 seconds vs the logarithm of the concentration for 2,3-DHBA, SRFA, phthalic acid, 1,2,4-BTCA and NaCl. The solid concentration was kept at 104 mg/L.

**References**

Fattahi A, Solouki T (2003) Using solution equilibria to determine average molecular weight of the Suwannee River fulvic acids Analytica Chimica Acta 496:325-337 doi:10.1016/s0003-2670(03)01010-9

International Humic Substances Society (2010) International Humic Substances Society. <http://www.humicsubstances.org/>. 2013

Ohshima H (1995) Electrophoretic mobility of soft particles Colloids and Surfaces A: Physicochemical and Engineering Aspects 103:249-255 doi:<http://dx.doi.org/10.1016/0927-7757(95)03293-M>

Tipping E (2005) Cation binding by humic substances. Cambridge Environmental Chemistry Series. Cambridge university press, United Kingdom

Topping DO, McFiggans GB, Coe H (2005) A curved multi-component aerosol hygroscopicity model framework: Part 2 – Including organic compounds Atmos Chem Phys 5:1223-1242 doi:10.5194/acp-5-1223-2005
